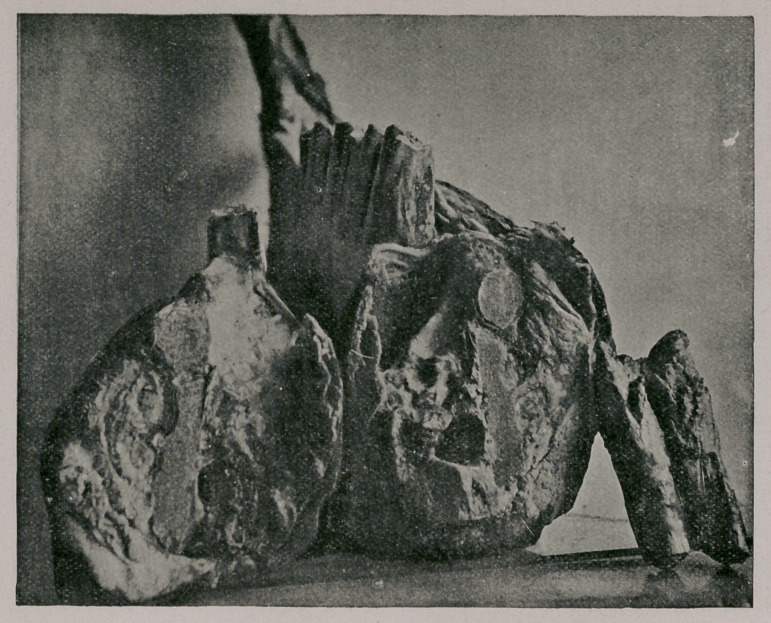# The Pathology of Actinomycosis, with Record of Cases and Experiments

**Published:** 1889-04

**Authors:** George A. Bodamer

**Affiliations:** Physician in Chief to the German Hospital, Philadelphia; Late Assistant to the Pathological Laboratory, University of Pennsylvania


### THE JOURNAL

— OF —

### COMPARATIVE MEDICINE AND SURGERY.

VOL. X. APRIL, 1889. NO. 2.

### ORIGINAL COMMUNICATIONS.

### ART. IX.—THE PATHOLOGY OF ACTINOMYCOSIS, WITH RECORD OF CASES AND EXPERIMENTS.

BY GEORGE A. BODAMER, M. D., B. S.,

*Physician in Chief to the German Hospital, Philadelphia; Late Assistant to the Pathological Laboratory, University of Pennsylvania.*

The present paper is a record of personal observations on Actinomycosis which I commenced in 1882 in the Pathological Laboratory of the University of Pennsylvania under the guidance of Dr. H. F. Formad. Early in 1883 I demonstrated to the Laboratory class the Actinomyces, or the radiating fungus[1] peculiar to Actinomycosis, and my inaugural thesis consisted of a record of some of my cases of that disease and of the description of the fungus discovered by myself. It was awarded the first prize in 1884 by the Medical Faculty of the University.

Hence I am probably entitled to priority in demonstrating the Actinomyces in the United States. It appears, though, that since I made my demonstration of the pathology of Actinomycosis it has received but little attention until recently, although the disease itself has been known for a long time as one peculiar

[1] Dr. Belfield, of Chicago, and Professor Law subsequently described the Fungus in American cases.

to cattle. It is known in this country under the name of "Swelled Head," and affects usually the jaws of the *Graminivora*, particularly the Ox tribe; less frequently it affects the tongue, the malady being then designated as "Wooden Tongue."

The disease has been recognized as a malignant one and metastasis to internal organs is known to occur. It was also recognized to be contagious or infectious, as it appeared to be transmissible from one animal to the other by cohabitation.

As to the Pathology of the disease different authorities in former times expressed most diverse opinions, and the affection was designated as Osteo-sarcoma, Cancer, Scrofula, Tuberculosis, Osteo-porosis, Hyper-ostosis, Fungoid Stomatitis, Sarcomatitis, etc. Some authors looked upon it as a kind of Glanders or Farcy of the Ox tribe.

Only since Bollinger more closely studied the disease in 1877 and designated it Actinomycosis, on account of a fungus, the Actinomyses, found to inhabit this lesion, a uniform conception of the nature of the disease was formed by the veterinarians of all lands, and the definition and name as given by Bollinger to this disease was generally adopted.

Ponfick, in 1879, was the first who described Actinomycosis in man, although the disease had been observed by Israel and described as chronic pyæmia one year previous. And as early as 1845, Langenbeck also described a case which was unquestionably Actinomycosis.

In the animal, the disease manifests itself as a primary disease of bones forming tumor masses, secondarily, giving rise to fistulous openings toward the exterior, and finally, metastasis to the internal organs occurs, particularly the respiratory tract where nodules are seen not distinguishable from miliary tubercles.

In man, however, the disease commences in the soft parts as fungoid suppurating granulations which ulcerate and finally proceed to the bones, inducing in the latter a caries or necrosis with everlasting chronic suppuration, and sometimes leading to exostosis or hyperostosis of the affected bones. Most commonly the disease seems to commence as a pleurisy which takes a chronic course, terminating in ulceration of the adjoining bones, *viz.* :—the vertebral column and the ribs. An interesting case of human Actinomycosis which was observed lately I will record in the latter part of this paper.

Unquestionably the disease must be classed with the group of granulation tumors, a group of new formations in which Virchow includes sarcoma, tubercle, syphilis, leprosy, lupus, glanders, etc.

The new formation of Actinomycosis which may be properly designated as "Osteo-myhomia," is histologically closely allied to sarcoma.

On account of a fungus discovered by Bollinger as above stated, inhabiting the lesions of the disease I am describing and to which Bollinger gave the name Actinomyces because of the radiating structure of this fungus, the disease is considered a parasitic one.

I have had the opportunity of seeing nine cases of Actinomycosis bones in Philadelphia, and one case in Berlin during my stay with Virchow in the summer of 1883. I made also some histological observations and cultures of the fungus and inoculation experiments on animals.

I have found, however, the fungus said to be peculiar to this disease in substances outside of Actinomykomatous lesions, and among other locations in ordinary caries of bone and teeth in man.

From these facts, and from the results of my experiments, I came to the conclusion that the Actinomyces are only concommitant and are diagnostic of, but probably not the cause of the disease.

CASE I.—Early in April, 1883, Mr. X., a butcher, whom I had requested to supply me with all kinds of diseased products in animals, as I was pursuing some comparative Pathologico-anatomical studies, informed me that he had an ox affected by a disease which he called "Swelled Head."

Upon my request I was permitted to be present when the animal was slaughtered.

Mr. X. informed me that animals thus affected are found once in a while, but that it was customary in his establishment, as well as in other droveyards, not to refuse such animals, unless particularly noticed by the authorities.

The animal in question was twelve years old and showed signs of having worked hard; it was sold on account of the lesion in the jaw. The lesion referred to consisted in a swelling of the right sub-maxillary bone, the tumefaction nearly altogether protuding outwards and downwards. The left side of the lower jaw as well as the upper jaw, the tongue and pharynx and the mucous membrane of the mouth with exception of one point to be referred to, were perfectly normal. The swelling was due to a tumor painful on pressure, perfectly immovable and consequently affecting the bone as well as the surrounding soft parts; in size ten inches in length, four and one-half inches high, and three and one-half inches in width. In the skin covering the tumor and in the portion corresponding to the lower border of the maxilla affected, there were five ulcerated spots varying in size from one fourth of an inch to one inch in diameter, which proved to be openings through which the probe could be introduced into the tumor to a depth of from one to four inches.

After the animal was killed, the skin removed, and the lower jaw detached from the rest of the head, closer examination showed the following: The swelling was a uniform oval tumor affecting the inferior-maxillary bone on the right side in the region corresponding to the molar teeth without affecting the latter, and extending as stated above chiefly downward and outward.

To the touch the tumor was quite hard in most places, while in others it easily yielded to pressure showing that it was partly bony in character and partly made up of soft material. The teeth as stated were in perfectly good condition. Externally near the gums where the tumefaction began, there were a number of whitish patches or surfaces of indurated tissue which were continuous with the tumor but no ulceration was found to open in the cavity of the mouth.

The appended photograph represents the specimen in its appearance, or rather its shape when fresh, reduced in size.

*Plate I.*—Represents the lower jaw detached and separated from the animal under consideration; it gives the aspect viewed from above. On the right side is seen the tumor mass in its actual shape and appearance, and relations to the part. On the upper surface along the outer border of the teeth is seen a narrow depressed surface somewhat abraded and bordered by the remnants of the detached buccal mucous membrane.

*Plate II.*—Represents the same specimen viewed from below. At the periphery of the specimen are distinctly seen some nodes and openings.

The tumor was then sawed transversely in several portions to study its structure. It was seen that a new growth resembling sarcoma involved the spongy tissue of the bone, invading and destroying most of the cancellated and part of the compact substance of the bone, the latter being destroyed completely in some places, and in other places leaving only a sheet as thin as paper. The new growth involved also the soft parts; the muscles, connective tissue, and even the skin, and giving rise to the sinus leading downwards as described above. Very instructive preparations were obtained in those sections where a tooth had been sawed through in its longitudinal axis, exhibiting its roots and socket. It was now seen that there were fistulous openings leading to the socket of the tooth not detectable from the outside, and which appeared as if healed up ulcerated passages led directly to the diseased parts. The healing of these fistulæ had been accomplished probably on account of the drainage established to the exterior in the lower part of the tumor through the openings in the skin.

The cut surface of the tumor presented further the following peculiarities. On scraping, there was a milky ichorous juice containing a few yellowish granules, in size from a poppy seed to a small pea. To these granules I will refer later. Throughout the whole cut there could be felt islands and streaks of bony structure alternating with the tumor mass which presented various appearances, in some portions being quite hard and fibrous. In others soft and friable, and in still others gelatinoid and even puriform.

Throughout the whole new formation, but more so in the softer parts, could be seen the yellow granules referred to. As a striking peculiarity, I may note that these yellow granules appear to multiply post mortem, when the specimen is preserved in its fresh state, for instance, in a cool place for a number of days. After scraping the yellow granules away, some new ones would again appear on the surface projecting from the tissue, in the lapse of a day or two.

I omitted to state that the animal from which the specimen was taken was examined carefully in all its parts, and none of the organs or tissue of the rest of the body was found to be diseased, so that the disease was evidently a local one.

In all parts of the tumor, in the firm as well as in the soft, and in the purulent exudation of the liquifying parts, I observed the yellow granules referred to above. These granules proved to be made up mainly of a branching or radiating fungus which I subsequently identified with the Actinomyces of Bollinger and the lesion itself as Actinomycosis.

CASE II.—In June of 1888, while working in Professor Virchow's Pathological Institute at the Charité, in Berlin, I saw a specimen of bone disease similar to that described in Case I.

I learned that Dr. Israel, one of Virchow's assistants, was studying up this affection, and was pleased to hear that I was not mistaken in my observation, namely, that the specimen under consideration was one of Actinomycosis Bovis.

Dr. Israel kindly demonstrated to me his own preparation of the fungus obtained from the lesions, which, however, did not correspond exactly with the organisms detected by myself in the American specimen. But in my further studies I learned that Israel's fungi were identical with mine, and merely represented different stages of the same growth.

The specimen in question was from a cow of the Municipal Drove Yard (Berliner Stadt Vieh-hof),[1] the left lower jaw of which was affected by a large tumefaction in the region of the molar teeth, the new growth also affecting the latter. The tumor was about twelve inches long and five inches in its greatest width, oval in shape, with nodular uneven surface.

On sawing the tumor open the appearance presented was in many respects very similar to that described in the Philadelphia specimen, only that the teeth and the alveolar processes were much displaced by the invading new formation. Here, also, the spongy portion of the bone was preeminently invaded and, as well as some of the soft parts, substituted by large, nodular, sarcoma-like, or fibroid masses. In other portions there were abscesses and fistulæ filled with a puriform semi-liquid mass, and cavities filled with luxuriant bony exostosis, and with necrosed bone particles, in addition to an

[1] As far as I remember, animals affected by Actinomycosis, which is quite a common disease in Germany, are not rejected by the German Sanitary Authorities. At this point I also would like to draw the attention to a rule adopted by the Berlin Municipal Board of Health in relation to the detection of infective diseases in animals used for food. As a measure to prevent, for instance, the sale of pork infected by trichiniasis, the butchers are induced to surrender such animals to the health office under conditions very profitable to them. The owner of the affected carcass is paid the full value of the animal and all extra expenses incurred from its surrender, thus earning at once the money which he otherwise would receive from the retail sale of the meat. Such measure is besides enforced under penalty of the law.

ichorous liquid. Throughout the new formation there were also seen numerous granules, such as I described in my first case, and made up of many bunches of the Actinomyces fungus. Some of these granules were yellowish, others rather white or gray, and distinctly calcified, so that the pulpified new growth felt gritty, as if mixed with sand.

Some of the tumor formations extended beyond the boundary of the bony framework and invaded extensively the surrounding soft parts.

The microscopical appearance of this Actinomykoma, the description and culture of the fungus, as well as the details of other histological studies made in the Berlin laboratory in connection with this disease, I will give collectively later on.

CASE III.—The specimen from this case was observed, as in Case I, during life. It was taken from a bullock, six years of age, in the Philadelphia slaughter house of Mr. X.

The part affected in this case was also the lower jaw, no other organ in the body of the animal being affected, except the lungs, which were partly collapsed and partly enphysematous. I made a careful examination of all the viscera, the spinal column, and of some of the bones. It may, however, be noted that the animal was intensely emaciated. The Actinomycosis was in this case left-sided.

The tumor, as in the other cases described, appeared to have sprung from the region of the alveolar processes, extending into the spongy portion of the bone, but only partially and in some places, so that a part of the narrow cavity was not affected.

In size the tumor was smaller than in Cases I and II The tumefaction, including the bony framework, did not exceed three inches in width in its longest diameter and six inches in length. The new growth invaded in some places also the soft parts, inducing several fistulous openings.

A peculiar feature was presented by some of the teeth, especially the fourth molar tooth. Here was seen a fistulous opening leading from without into the socket of the tooth, and thence communicating with the new formation within the spongy portion of the bone. The tooth appeared quite loose, and upon removal presented appearances such as shown in Plate III. There were at the root a number of nodules of the new formation, about the size of a pea, or smaller, which upon examination proved to consist of actinomycomatous tissue. Upon scraping away these new formations the roots were seen roughened, exhibiting some bony excresences or small hyperostosis.

The floor and the lateral walls of the socket appeared on the contrary rather necrosed, and besides showed depressions corresponding to the excresences upon the tooth. See Plate III. That these appearances were abnormal was proved on examination of the tooth and socket of the corresponding normal side, which was examined for comparison. The other appearances and characters of the new formation were precisely the same as in Case I, only they were much less extensive, exhibiting also less softening and much less ichorous or liquifying changes. Actinomyces granules were found here and there scattered throughout the affected tissue, but these were not so abundant as in the specimens before described.

CASE IV.—The specimen was from the right jaw of a cow and obtained from the Philadelphia Abattoir. I could not obtain information concerning the age of the animal from which it came, nor whether there were any other lesions in the body. The specimen was also somewhat mutilated in its soft parts and broken or cut in half with an ax; still it represented an evident and typical case of actinomycosis.

The tumor invasion in this specimen was very extensive, although apparently not invading the teeth, which were left intact in the specimen. (Some of the teeth were broken out and the alveolar processes damaged when I received the specimen.) Otherwise the specimen was in every respect similar to that which I obtained in Berlin, and with lesions perfectly identical with those of the latter. Actinomyces granules were found abundantly, many of them being calcified.[1]

THE MORPHOLOGY OF THE LESIONS.

*Microscopical studies of the specimens from the above cases.*—From the character of the specimens, their naked eye appearances and the fungus which I detected in clusters making up the mentioned yellow minute bodies, I was fully aware that I dealt with lesions from a disease designated as Actinomycosis. All the cases, however, which came under my observation respresented only the local lesion of this disease.

It is well known now, that the disease also generalizes itself, the primary new growth spreading by continuity of structure and, by metastasis, affecting internal organs. This genralization appears, however, to be the exception rather than the rule from what I could learn in my own studies. The new growth which I described above and of which I am about to give the histological details, must unquestionably be classed with the tumors, being closely allied to Osteo-sarcoma, with which it was formerly identified. It might be properly called *Actinomycoma*. All the cases including that met in Berlin, are in all their chief features perfectly analagous.

The microscopical studies also reveal their full identity so that the description of one, will suffice for all. From the naked eye appearance, and from physical properties, it was seen that there

---

[1] Since writing this paper Mr. X. sent me a new and magnificent specimen of Actinomykoma of ox (fifth case). The tumor in this case is of the size of a man's head and affects the left upper jaw and evidently also the orbit. Some of the teeth are necrosed and fistulous openings lead to the interior of the tumor. The tumor otherwise appears to be identical with the specimens described above. A difference in relation to the affection of the teeth may be noted between my cases and those of Dr. Belfield of Chicago. Dr. Belfield does not describe any lesions of the teeth in the animals affected.

were at least five distinct morphological constituents of which an Actinomykomatous tumor was made up :—

1st. The tissues forming the boundary of the tumor.
2d. The bony frame-work.
3d. The new-growth proper in its solid parts.
4th. The liquified products.
5th. The yellow granules scattered throughout the whole of the above constituents.

1st. *The tissues making up the boundary of the tumor.*—These were not distinct. The new-growth gradually shading off into the bony parts or else the integuments formed the boundary in such places where the bone was completely absorbed. The surrounding soft parts were as a rule much atrophied, and, as the microscope showed, infiltrated by cells from the new-growth. In some specimens parts, of the tumor masses were observed to be encircled by dense masses of connective tissue. These connective tissue bands were seen on section sometimes to penetrate into the new-growth. Some parts of the tumor were occasionally seen to project outside as lobulated fungoid masses. As a rule, however, the surface of the tumor was smooth. Contrary to my expectations I found the yellow granules composed of masses or tufts of fungi, even in the most peripheral portions of the tumor. The capsule showed one large and several small clusters of the fungus imbedded between the connective tissue fibres.

2d. *The bony framework*, being similar to myeloid sarcomata, it appears to me that while the new growth is formed within the bone structure and at the expense of the latter, the bone also grows by opposition. The bony framework forms round or irregular spaces of various sizes, and while these spaces enlarge the bone tissue seems also to grow, and even to send bony excrescences or exostoses into the cavities filled by the new-growth ; I thought it possible that these apparent exostoses might be remnants of unabsorbed new-formed bone tissue. Often spicules of bones are met with in the soft tumor masses. Upon making section of the bony excresences I found that they had the structure of young callus tissue. Large osteoclasts such as found in the repair of bone or callus formation are seen to make up the bulk of these structures. The cells are imbedded in a granular matrix, such as is common in young callus tissue before complete ossification sets in. Here and there are seen islands of mature bone tissue.

From the structural appearances of these bony excrescences it is evident that they are not remnants of bone tissue, but rather new formations. This tends to show that the new formation of bony or callus tissue is a part of the tumor formation in actinomycosis.

3d. *The new growth proper in its solid parts.*—I have here reference to the nodular formations usually lobulated in character which formed the softer portions of the tumor. Sometimes indeed they are nearly semi-liquid ; it is these parts which by means of fatty degeneration gives rise to the liquid parts of the tumor.

The appearance of this substance is white, often glistening, sometimes of milky appearance, in other places it is quite congested and red, and even specks of ecchymosis are occasionally seen.

Under low magnifying power a section presents granular appearance through which bands of connective tissue pass in various directions dividing the tissue into many partitions. In some places the granular appearance predominates particularly so in the centre of the nodes, while at the periphery the structure has nearly a fibroid appearance. Under higher magnifying power the appearances of the new-growth suggests very much Sarcoma, either in its round or spindle cell variety ; in other portions suggesting alveolar sarcoma, and in still other dense masses of connective tissue are seen into which the younger cells gradually merge.

4th. *The liquifying products*, or a creamy liquid derived from the necrotic changes of the solid parts, were quite abundant in nearly every specimen ; the centre of the larger nodes and sometimes the whole of a large node had undergone this change.

Under the microscope a small particle of the semi-liquid material, well teased, often showed quite distinct alveolar connective tissue, intermingled with leucocytes.

5th. *The yellow granules* scattered throughout the whole of the above described constituents of the Actinomykoma are clusters of the fungi which inhabit this new growth.

The fungus forms a necessary part of the anatomy of the new growth, or at least is extremely common. All my cases contained the fungus. On the other hand, if the fungus had not been present the new growth would have to be called sarcoma, rather than Actinomykoma.

No definite distribution of the fungus can be made out, the yellow granules, which represent and which are easily distinguishable by the naked eye, are as a rule seen everywhere throughout the specimen.

The fungus is found more numerous in the liquifying parts, and here more often in small fragments, so that if a drop of liquid is taken and examined microscopically a large amount may be seen. In most instances masses containing the fungus were calcified.

INOCULATION EXPERIMENTS.

That the disease is transmissable from one animal to another, there is now no reason to doubt, as the fact has been experimentably demonstrated by Johne & Ponfick. The experiments of these and others had previously failed, probably because the inoculation material was too old and had undergone change.

Johne[1] subsequently employed quite fresh material, and was successful in three out of four experiments—the animals being too calves, a cow, and a foal. The latter remained unaffected. The calves were inoculated subcutaneously behind the lower jaw, and elsewhere, and a small quantity of the same material which was derived from a tumor of a living cow was also introduced into the peritoneal cavity. In one case death took place forty days after inoculation. The calf having lost its appetite, became emaciated and debilitated, and then succumbed. At the seat of inoculation, as well as in the abdomen, Actinomycosis was markedly developed. In the second case death took place 114 days after inoculation, and the results were found to be as marked as in the other instance.

The third case was a pregnant cow, which gave only a small quantity of milk. This animal was inoculated through the milk duct of the teat. The inoculation was in a few days followed by inflammatory œdema, which soon became developed into phlegmonous mastitis. Without any treatment the inflamation subsided, but there remained a small hard swelling which increased so much that in three months the quarter of the gland was double its normal size, and felt like a hard fibroma.

---

[1]Johne. Actinomykosis Bericht übár d. Veterinärwesen im Konigreich sachsen, 1881.

No milk was secreted. The cow was killed 133 days after inoculation, and in the udder were discovered all the signs of Actinomycosis ; diffuse fibroma, with, variously located multiple spongy fibro-sarcomata, the interspaces of which contained the characteristic nodules or "granulation tissue," enclosing the fungus. Ponfick[1] did not succeed in inoculating dogs or rabbits. He endeavoured to produce the disease in cattle by feeding them with infective material in the form of fresh nodules, but the results were negative. By subcutaneous inoculation and intra-venous injection, however, he was completely successful, and the lesions of the former were similar to those in Johne's cases. Injection of the material into the jugular vein produced in the course of two or three months typical new-formations in the lungs. The details of these experiments are very interesting, but too voluminous to be quoted in full. But they by no means conclusively prove the identity of the artificial with idiopathic disease, save the presence of the Actinomyces which was found naturally because it had been introduced.

There is no record of instances which might tend to show that the disease may be accidentally transmitted.

Ponfick relates the case of a woman, thirty-four years of age, who was attacked by the disease after having been for several years employed as a servant, in which position she was frequently among sick cattle, and these were affected with what the veterinary surgeon who attended them called "wurm," the popular name in Germany for the malady under consideration. This is the only case of probable transmission recorded.

To test the specific properties of the Actinomyces fungus or materials containing it, I made the following experiments, strict antiseptic precautions being taken while the animals were kept separated and under the best hygienic conditions :—

[1] Ponfick. Die Actinomykose der menschen, eine neue infections krankeit. Berlin, 1882.

### TABLE OF EXPERIMENTS.

| No. of Experiment. | Date of Inoculation. | Animal. | Inoculation. | The Effects Produced and Termination. | Result of Post-mortem and Microscopic Examination. | Remarks. |
|---|---|---|---|---|---|---|
| 1 | Nov. 28, 1883. | Rabbit. | Inoculation made in left sub maxillary bone and on the spine in the lumbar region, cutting down to the bone. Inoculation material taken from the soft part of an Actinomykoma of Ox from Case III. | Wound healed at once. The rabbit did not appear ill, but on the third day swelling appeared on the seats of inoculation which on the fifth day formed ulcers and discharged pus. Subsequently cheesy lumps appeared which persisted up to the death ot the animal, which took place forty three days after inoculation. | Animal was emaciated, cheesy humors of the size of pigeon's eggs at seat of inoculations but not complicating bone ; Actinomyces found abundant in the cheesy mass but not in the surrounding parts. Lungs full of miliary tubercles also *liver*, lymphatic glands enlarged. Rest of organs normal. | It must be noted that no real actinomyces lesion was produced, but only a cheesy node, which served as culture soil for the fungus introduced. Tuberculosis was the result in internal organs mentioned. |
| 2 | Nov. 28, 1883. | Rabbit. | Inoculation made in angle of jaw and in spinal column in lumbar region with material from Case III of Actinomykoma. | Wounds healed and formed cheesy masses of the size of a walnut at seat of inoculation. | Rabbit died Jan. 10, '84. Autopsy showed the lesions to have remained local, being expressed by the cheesy mass. Actinomyces was found, but only peripheral in the wound, while the cheesy mass proper was free from them. | . . . . . . . . . . . |

| | | | | | | |
|---|---|---|---|---|---|---|
| 3 | Nov. 28, 1883. | Rabbit. | Inoculation in thigh and angle of jaw. | Wounds healed and formed cheesy masses of the size of a walnut at seat of inoculation. | Rabbit died Jan. 15, '84. Otherwise same as experiment 2. | . . . . . . . . . . . . |
| 4 | Nov. 28, 1883. | Rabbit. | Inoculation made in spine at the neck. | Wound healed without result except small cheesy mass. | Rabbit died Feb. 1, '84. Organs full of miliary tubercles, nothing else peculiar. No Actinomyces found. | . . . . . . . . . . . . |
| 5 | Nov. 28, 1883. | Rabbit. | Inoculation made in the neck on the spine as in the last case. | Wound healed, cheesy mass remained. | Rabbit died Feb. 15, '84. All organs full of miliary tubercles. No Actinomyces, | . . . . . . . . . . . . |
| 6 | Jan'y 8, 1884. | Dog. | Inoculation made on spinal column cutting down to the bone and also scarifying the bone. The matter used was taken from soft parts making sure that it contained the fungus. | Wound did not heal, but after three days became phlegmonous and indolent. The skin and subcutaneous tissue very much indurated. The deep structure, *i. e.*, the muscle and bone seemed to be very much affected, there is swelling of the parts and hardness. The contents of the swelling is drained through a fistulous opening which throws off continuously a puriform liquid. | The dog is otherwise well and is kept for further observation. The pus drained from the Fistulæ contains Actinomyces. | . . . . . . . . . . . . |

TABLE OF EXPERIMENTS—(*Continued*).

| No. of Experiment. | Date of Inoculation. | Animal. | Inoculation. | The Effects Produced and Termination. | Result of Post-mortem and Microscopic Examination. | Remarks. |
|---|---|---|---|---|---|---|
| 7 | Jan'y 8, 1884. | Large cat. | Inoculation made with same material as in case V, in the thigh and angle of jaw. | Wounds healed and an indurated node formed subcutaneously which at first enlarged, but now is gradually subsiding. | Observation is continued. | . . . . . . . . . . . |
| 8 | Jan'y 8, 1884. | Small cat. | Inoculation made with same material as in Case V in the thigh. | Wound healed rapidly and cat became emaciated. Cat died Feb. 28, 1884. | Autopsy showed a miliary nodular eruption in all its organs. These nodes did not contain fungi of any kind and proved to be genuine tuberculosis. | . . . . . . . . . . . |
| 9 | Jan'y 8, 1884. | Cat. | Inoculation with same material as in Case V in angle of jaw. | Wound did not heal and a fistular opening communicating with an abscess cavity below. | Pus from fistulæ contains Actinomyces. Observat'n continued. | . . . . . . . . . . . |
| 10 | Nov. 30, 1883. | Dog. | Inoculation in jaw with material from Case III of Actinomycosis bovis. | No effects, &c. | Died Dec. 20, 1884. No lesions found. | . . . . . . . . . . . |

| | | | | | | |
|---|---|---|---|---|---|---|
| 11 | Nov. 30, 1883. | Rabbit. | Same as last. | Matter from fistulæ for a few days showed the fungus when gradually all healed up. | Died Jan. 12, 1884. No lesions found. | . . . . . . . . . . . . |
| 12 | Nov. 30, 1883. | Rabbit. | Inoculation with cultured fungi obtained from infusion of corn, made in spinal column of neck, scraping the bone. | Wounds healed on 3d day but soon cheesy lumps developed, identical with those lesions met with in the inoculation of fungi derived from the actinomycotic lesions. Animal died Jan. 20, 1884. | Autopsy showed no internal lesions; the bone at seat of inoculation was found necrosed. The swelling consisted of an organized mass made up of connective tissue and lymph cells in state of cheesy degeneration and containing the fungus. | The fungi obtained from the infusion stated were in all respects identical with the actinomyces found in the bovine disease. |
| 13 | Nov. 30, 1883. | Dog. | Same as above. | Wound healed. | Animal was killed March 1st, but no lesions found on autopsy. | . . . . . . . . . . . . |

My experiments show that inoculation of animals with materials from Actinomycosis was followed, so far, by a formation of marked lesions in six out of thirteen animals.

One of the positive experiments showed the identity in the effects of fungi resembling Actinomyces but found in matter outside of diseased products (in corn). But the experiments, I confess, are not altogether satisfactory, not being repeated in sufficient number. One thing they prove, however, that the lesions produced by inoculation do not differ from tuberculosis except in containing instead of the tubercle bacillus the Actinomyces fungus or no fungi of any kind. The presence of the Actinomyces is not suprising as I already expressed above, as that fungus was introduced.

Control experiments with other than Actinomycosis material were not made because I had witnessed in the investigations of Dr. Formad very numerous experiments with simple irritants to terminate in precisely the same manner as my above stated experiments, and furthermore, I have seen that the lesions of induced tuberculosis have precisely the same appearance and manifestations as those obtained in my experiments with material from Actinomycosis.

BIBLIOGRAPHY.

Langenbeck in 1845 quoted by Israel. Virchow's Archiv. Vol. 74, p. 50, 1878.

*Rivolta.*—"Sarcoma fibroso al bordo inferiore della branca Mascellare Sinistra nel bue." Il Medico Veterinario. Turin, 1868, p. 125.

*Rivolta.*—"Del cosi detto Farcino o Moccio dei Bovini e della Cosi detta Tuberculosi o mal del Rospi (Trutta) della lingua dei Medisimi Animali." Giornale dé Anat. Fisiol. e Patol. degli. Animali. Pisa, 1875, p. 198.

*Perroncito.*—"Osteosarcoma della Mascella anteriore e posteriore nei Bovini," etc. Enciclopedia agraria Italiana, etc. Turin, 1875, Part 6, p. 569.

*Bollinger.*—"Uebereine neue Pilzkrankeit beim Rinde." *Centralblatt f. d. Medic. Wissenschaften.* Vienna, 1877, No. 27, *Deutsche Zeitschrift fur Thier-medicin.* Leipzig, 1877, p. 334.

*Harz.*—"Actinomycosis bovis einer neuer schimmel in den Geweben des Rindes." Jahresbericht der Münchener Schule, 1877–78.

*Siedamgrotzky.*—"Actinomykose." Bericht über d Veterinärwesen im König. Sachsen für d. Jahr, 1877. Dresden, 1878, p. 28.

*Israel.*—"Neue Beobachtungen auf dem Gebiete der Mykosen des Menschen." Virchow's archiv für Path. Anat. Berlin, 1878, p. 15.

*Rivolta.*—"Sul cosi detto mal del rospo del Trutta e sull' Actinomyces bovis di Harz." *La Clinica Veterinaria.* Milan, 1878.

*Perroncito.*—"L'Actinomyces bovis (Harz) ed i sarcomi nei bovini." Annali della Reale Accad. d' Agricultura. Turin, 1878.

*Johne.*—"Epulis vom Rinde mit Actinomyces Boum." Bericht über die Veterinärwesen im König. Sachsen für die Jahr 1878. Dresden, 1879, p. 26.

*Ponfick.*—"Ueber eine eigenthumliche Form prævertebraler Phlegmon." *Berliner Klin. Wochenschrift*, Berlin, 1879. *Breslauer Arztliche Zeitschrift* 1879. Breslau.

*Rivolta.*—"Sopra un nuovo micromicete del Cavallo." Piacenza, 1879, p. 145.

*Perrioncito.*—"Ueber den Actinomyces bovis und die Sarkome der Rinder." *Deut. Zeitschrift fur Their-medicin.* Leipzig, 1879, p. 33.

*Johne.*—"Actinomykosis." Bericht über die Veterinarwesen im König. Sachsen für die Jahr 1879. Dresden, 1880.

*Israel.*—"Neue Beiträge zu den mikotischen Erkrankungen des Menschen" Virchow's Archiv. Berlin, 1880.

*Ponfick.*—"Ueber Actinomykose des Menschen." *Breslauer Arztlich. Zeitschrift.* Breslau, 1880, pp. 141, 155.

*Rosenbach.*—"Zur Kentniss der Strahlenpilzer-Krankungen beim Menchen." *Centralblatt fur Chirugie.* Leipzig, 1880. No. 15.

*Rabe.*—"Casuistiche Beiträge zur Geschwulstlehre." *Wochenschrift fur Thierheilhunde and Viehzucht*, 1880. No. 4.

*Ponfick.*—"Ueber Aktinomykose des Menchen." *Berliner Klin. Wochenschrift.* Berlin, 1880. P. 660.

*Nosotti.*—"Sul cosi detto mal del Forbice." *La Veterinaria, Casalmaggiole.* 1880. pp. 342. 453.

*Partsch.*—"Zwei Fälle von Actinomycosis." *Breslauer Arztlich Zeitschrift.* Breslau, 1881. P. 78.

*Johne.*—"Die Actinomykose oder Strahlenpilzerkrankung eine neue Infectionskrankeit." *Deutsche Zeitschrift fur Their-medicin.* Leipzig, 1881. p. 141.

*Csokor.*—"Die Strahlenpilz-Erkrankungen, Actinomykosis." *Allgemeine Weiner Mediz. Zeitung.* Wien, 1881. No. 43.

*Ponfick.*—"Die Actinomykose der Menschen, eine neue Infectionskrankheit." Berlin, 1882.

*Bizzozero.*—"L'Actinomicosi, Una nuova Malattia da parassiti Vegetali." *Gazetta delgi Ospitali.* Milan, 1882.

*Micellone e Rivolta.*—"Di una nuova specie di micromicete e di Sarcoma nel Cavallo, Giornale d' Anat. Fisiol, e Patol. degli Animali." Pisa, 1882.

*Vachetta.*—"Osteochondrosarcoma macrocellulaire con Actinomyceti alla Mandibola inferiore d'un cane." *La Clinica Veterinaria.* Milan, 1882. P. 226.

*Lindquist.*—"Aktinomykos, en infectionssjukdom hos nötboskap och Svinkreatur." Tidskrift för Veterinär-Medicin och Husdjursskötsel. Stockholm, 1882. P. 165.

*Putz.*—"Actinomykose die Seuchen und Herdekrankheiten unserer Hausthiere." 1882. P. 592.

*Pflug.*—"Ueber Actinomykosis." *Berliner Klinischen Wochenschrift.* 1882. No. 3. *Oesterriechische Vierteljahresschrift fur Wissenschaftliche*

*Veterinarkunde*, 1882, Band LVIII, Heft 1. *Centralblatt fur die Medicin, Wisensschaft*, 1882, No. 14.

*Johne.*—"Actinomykose der Zunge." Bericht über d. Veterinärwesen im Königr. Sachsen pro, 1881.

*Belfield.*—"Actinomycosis." *Medical News*, Nov. 24, 1883, p. 569.

*Law.*—"Actinomycosis in Animals." *Medical News*, p. 695, Dec. 22, 1883.

*Muller, E.*—"Ein Fall von geheilter Actinomykose." "Meth. a. d. Chir. Klinie. Tubingen," 1883. pp. 17 and 19.

*Lemann, A.*—"Sull Actinomycosi del Peritonea dei Viscera Addominali nell umano." *Med. Jahrb*, 1883, p. 477.

*Shattock, S. G.*—"Two cases of Actinomycosis in man." Tr. Path. Soc. London, 1884, Vol. XXVI, pp. 254–261.

*Bang, B.*—"Die Strahlenpilzerkrankung (Actinomykose)." *Deutsche Zeitschrift f. Thiermed.* Leipzig, 1884, p. 294.

*Meyer, D.*—"Sur un cas d'Actinomycose chez l'Homme." *Gaz. Med. de Strasburg*, 1884.

*Muller, E.*—"Ein Fall von geheilter Actinomykose." *Med. Cor. bl. des wurtbg Aerzt Ver.* 1884, p. 188.

*Calvin, J.*—"L'Actinomycosis" *Cac. med. de. Granada*, 1884, pp. 298–328.

*Treves, W. K.*—"One case of Actinomycosis." *Lancet.* London, 1884, p. 107.

*Fichler, Albert.*—"Beitrage zur Lehre von der Actinomykose." Halle, a S. 1884,

*Mauri, F.*—"Contribution à l'Étude de l'Actinomycose." *Rev. Med. de Toulouse*, 1884, pp. 617–629.

*Wolf, J.*—"Ueber einen Fall von Actinomykose." *Breslau aerztl Ztchft*, 1884, p. 284.

*Palagi, A.*—"Caso di rantolo in un vitello prodotto da Actinomicosi. esofagea." "Gior di anat. fisiol patol. d animali." Pisa, 1884, pp. 312–314.

*Dunker, H. C. J.*—"Strahlenpilze (Actinomyces) im Schweinefleisch." *Zeitschr. fur Mikr. und Fleischschau.* Berlin, 1884, pp. 17–19.

*Florkiewiez, W.* "Actinomycosis." Warsaw, 1885, pp. 911, 938, 955, 984.

*Wolff, J.*—"Ueber einen Fall von Actinomykose." *Jahresbr. der schles. Gesselsch. f. Vaterl. Kult.*, 1884. Breslau, 1885, pp. 113–121.

*Magnussn, L.*—"Beitrage zur Diagnostik und Casuistic der Actinomykose." Kiel, 1885.

*Satterthwaite, T. E.*—"Actinomycosis in Man and Animals." *Quar. Bull. Clin. Soc. N. Y. Post-Grad. Med. School and Hospital.* N. Y., 1885, p. 160.

*Bostion.*—"Verhandl. d. Cong. finnen med." Weisbaden, 1885, pp, 94–99.

*Murphy, J. B.*—"Actinomycosis in the Human Subject." *N. Y. M. J.*, 1885. pp. 17–19.

*Roger.*—"Ueber Actinomykose." *Aerztl. Int. bl.* Munchen. Vol. XXXI, p. 583, 1885.

*Ponfick.*—"Ueber Actinomykose ohn Actinomyces." *Breslau aerztl. Ztschr.*, 1885, p. 30.

*Wolff, J.*—Ueber einen Fall von Actinomykose." *Breslau aertztl. Ztschr.* 1885, p. 31.

"Artigalas De l'Actinomycose." *Rev. sau d. Bourdeaux*, 1885, pp. 75–77.

*Israel, James.*—"Klinische Beitrage zur Kenntniss der Actinomykose des Menschen." Berlin, 1885.

*Heller, A.*—"Ein Fall von Actinomykose unter den Bilde einer acuten Infections Krankheit verlaufend." *Deutsche Arch. f. Clin. Med.* Leipzig, 1885, pp. 372–375.

*Genersch, A.*—"Ueber Actinomykose." (Transl. from "Orrosihetil," 1885, Nos. 33 and 34.) *Pest Med. Chir. Presse.* Budapest, 1885, pp. 824–834.

*Ochsner, A. J.*—"Report of a Case of Actinomycosis." *Chic. M. J. and Exam.*, LIII, No. 6, pp. 1–3; also *J. Am. Med. Ass.*, Chicago, 1886, pp. 608–610.

*Scinassy, A.*—"Ein Fall von Actinomykose." *Pest Med. and Chir. Presse.* Budapest, 1886, p. 908.

*Firth, R. H.*—"On the Nature of the Actinomyces or Ray Fungus." *Indian M. J.* Calcutta, 1886, pp. 505–509.

*Moosburger.*—"Ueber die Actinomykose des Menschen." *Centralb. f. Chirurgie*, 1886, pp. 897–902.

*Kijewski, F.*—"Promienica Actinomycosis." *Kron. Lek.* Warsaw, 1886, pp. 313, 375, 427.

*Kinnell, G.*—"Actinomycosis in Cornwall." *Vet. J. and Ann. Comp-Path.* London, 1886, pp. 8–10.

*Soltmann.*—"Ueber Aetiologie und Ausbreitungsbezirk der Actinomy. kose." *Jahrb. f. Kinderh.*, 1886, pp. 129–139.

*Perroncito, E.*—"Inoculation d'Actinomycose Accidentellement Survenui a un Cheval." *Arch. Ital. de Biol.* Turin, 1886, p. 143.

*Jaudin, Joseph.*—"Etude sur l'Actinomycose de l'Homra et des Animaux." Lyon, 1886.

*Haddon, W. B.*—"Actinomycosis." *St. Thomas Hosp. Rep.*, 1884. London, 1886, p. 292.

*Wildermuth.*—"Ein Fall von Actinomykose." *Med. Cor. Bl. d. Wurt. arztl. Ver.* Stuttgart, 1886.

*Winter.*—"Ein Falle von Actinomykose bei einem Soldaten." *Deutsche mil. arztl. Ztschr.* Berlin, 1886, pp. 188–193.

*Acland, T. D.*—"Actinomycosis Hominis." *British M. J.* London, 1886, pp. 316–328.

*Partsch.*—"Einige neue Fälle von Actinomykose des Menschen." *Deutsche Ztschr. f. Chir.* Leipzig, 1885–6, pp. 497–529.

*Billroth.*—"Actinomykose." *Allg. Wien. Med. Ztschr.*, 1886, pp. 316–328.

*Roser, K.*—"Zwei Fälle von Actinomykose." *Deutsche Med. Wochenschr.* Berlin, 1886, pp. 367–371.

*Firth, R. H.*—"Note on a Case of Actinomycosis." *Indian M. J.* Calcutta, 1886, pp. 322–325.

*O'Neill, W.*—"A Case of Actinomycosis." *Lancet.* London, 1886, p. 342.

*Corneille.*—"Actinomycose." *J. d. Conn. Med. Pract.* Paris, 1886. pp. 289–297.

*Schirmer, A.*—"A Case of Actinomycosis Hominis." *Chicago M. J. and Examiner*, 1886, p. 354.

*Moosburger, Paul.*—"Ueber Actinomykose des Menschen." Tubingen, 1886.

*Oechsler, P. A.*—"Berträge zur Actinomykosis Hominis." Kiel, 1886.

*Kaarsberg. F.*—"Actinomykosis." *Nono. Med. Ark.* Stockholm, 1887, No. 22, p. 11.

*Raffa, A.*—"Athinomicosi addominale dell 'uome. Rio veneta di sc. med." Venezia, 1887, pp. 577–584.

*Rooseng, T.*—"Et Tilfaelde of Actinomykose." *Hosp. Tid. Kjobent.* 1887, pp. 889–895.

*Baranski, A.*—"Zur Farbung des Actinomyces." *Deutsche Med. Wochenscht.* Leipzig, 1887, p. 1065.

*Sommer.*—"Un caso di Actinomycosi." *Bolt. di clin.* Milano, 1887, pp. 393–400.

*Ullmann.*—"Ein Fall von Bauchactinomykose." *Anz. d. K K. Gesellschaft d. Aerzte.* Wien, 1887, p. 183.

*Claus, E.*—"Ueber die Localisation und geographische Verbreitung der Actinomykose beim Rinde in Bayern." *Deutsche Ztschr. f. Thier. Med.* Leipzig, 1887, pp. 290–300.

*Fisher, Ervın.*—"Beitrage zur Kenntniss der Aktinomykotischen Granulationen und der Histologie Aktinomykotischer Herde im Gehisne und seiner Haute." Tubingen, 1887.

*Koehler, A.*—"Ein Fall von allgemeiner und ein Fall von lokaler Actinomykosis." *Charite Ann.* 1885–1886. Berlin, 1887, p. 598.

*Skeritt, E. M.*—"Actinomycosis hominis." *Am. J. M. Sc.* Phila. 1887, pp. 75–88.

*Hebb, R. G.*—"Actinomycosis Hominis." *Lancet.* London, 1887, p. 313.

*Hebb, R. G.*—"A case of Actinomycosis Hominis." *Brit. M. J.* London, 1887, p. 331.

*Kapper, F.*—"Ein Fall von acuter Actinomykose." *Wein. Med. Presse*, 1887, p. 94–96.

*Munch, A. W.*—"Ein Fall von Actinomykosis hominis." *Cor. Bl. f. Schweiz. Aerzte.* Basel, 1887.

*Billroth, T.*—"Some remarks on Actinomycosis." *Ala. M. & S. Jour.* Birmingh., 1887, pp. 312–329.

*Braun, H.*—"Ueber Actinomykose beim Menschen." *Cor. Bl. der allgem. arztl. Ver. v. Tubingen.* Weimar, 1887, p. 37–51.

*Hebb, R. G.*—"A Case of Actinomycosis Hominis." "Proc. Roy. M. and C. Society." London, 1885—1887, p. 197.

*Hochnegg, J.*—"Zur Cauistick der Actinomykose des Menschen. *Wien. Med. Presse*, 1887, pp. 537–582.

*Majocchi, D.*—"Appunti clinici microscopici sopra un raro caso di Attinomycosi cutanea dell uomo." *At Med. Parmensi.* Parma, 1887, pp. 1, 67, 70.

*Carter, H. V.*—"Note on the apparent similarity between Mycetoma and Actinomycosis." Tr. M. & Phy. Soc. Bombay, 1887, pp. 86–90.

*Remy.*—Trois cas d' Actinomycosi chez la vache." *Ann. de Med. de Gand.* 1887, pp. 67–70.

*Hauken.*—"Een geval van Actinomycosis hominis." *Nederl. Tijdschr. v. Guersk.* Amst., 1887, pp. 494–496.

*Hochenegg, J.*—"Lur Casuistick der Actinomykose des Menschen." *Wein. Med. Presse.* 1887, 613,

*Mayer, M.*—"Beiträge zur Actinomykose des Menschen." *Prag. Med. Wochschr.* 1887, p. 161.

*Hochenegg, J.*—"Bauchwand Actinomykose." *Anz. d. K. K. Gesellschaft d. Aerzte in Wien.* 1887.

*Vou Sommers, G.*—Primo caso di Actinomycosi osservato in Napoli." *Rio di Med. e. Chir.* Napoli, 1887.

*Afanasjew, M. J.*—Ueber die Kleinische Microskopie und Bacteriologie der Actinomykosis." *St. Petersburg Med. Wochenschrift.* 1888, pp. 78–83.

*Baraz, R.*—"Actinomycosis transferred from Man to Man." *Purzgl lek.* Krakow. 1888, pp. 201–03.

*Bulhoes, O.*,—(E. P. S. Magathaes.) "Observagao de um caso de actinomycosa humana. *Brazil Med.* Rio de Janeiro, 1888, p. 12.

*Hartmann, R.*—"Ein Beitrag zur Actinomycosis hominis." *Allg. Med. Centr. Zeitung.* Berlin, 1888, pp. 869–71.

*Holst, A.*—"Et Tilfaelde of Actinomycosis hominis." *Nordsk. Mag. f. Laegevidensk.* Christiania, 1888, p. 326.

*Langhans, T.*—"Drei Falle von Actinomykose." *Cor. Blt· f. Schweiz. Aerzte.* Basil. 1888, pp. 329–371.

*Brazzola, F.*—"Sull istogenesi delle lisioni anatomo pathologiche dell' Actinomycosi. Modena, 1888, p. 165.

*Petroff, N. W.*—"Ein Beitrag zur Lehre von der Actinomykose. *Berl. Klin. Wochenschrift.* 1888, pp· 541-44.

*Afanagiff. M. J.*—"O Klinecheskoi mikroskopii e Bacteriologii aktinomikoza." St. Petersburg, 1888.

*Hebb.*—"A case of Actinomycosis hominis." *Westminister Hosp. Rep.* London, 1888, pp. 150–53.

*Israel, O.*—"Praparate mit Actinomycosis von der Leiche einer 44 jähr. Frau." *Berlin Klin. Worhenschrift*, 1888, p. 171.

*Braatz, E.*—"Zur Actinomykose, Leigbacterien im Horn." *St. Petersburg Med. Wochenschrift*, 1888, pp. 119–127.

*Munch, A.*—"Actinomycosis hominis. *Cor. Bl. f. Schweiz. Aerzte.* Bazel, 1888, p. 234.

*Schuchardt, B.*—"Ueber das Vorkommen des Strahlenpilzes (Actinomyces) und der Strahlenkrankheit (Actinomykosis) nebst Literatur der Actinomykose bei Thieren und Henschen." (Nach Hertwig's Berichten.) *Cor. Bl. d. allg. arztl. Ver. von. Thuringen.* Weimer, 1888, pp. 323–337.

*Bertha, M.*—"Uber einige bemerkenswerthe Fälle von Actinomykose." *Wien. Med. Wochenschr.* 1888, pp. 1181–1184.

*Glaser, Johannes.*—"Ein Beitrag zur Casuistick und klinischen Beurtheilung der menschlichen Actinomykose." Halle a. S. 1888.

*de Sampais Berros, Francisco.*—"Zur Pathologie und Therapie der Actinomykose beine Thiere und Menschen." Aachen, 1888.

*Eve, F.*—"Actinomykosis." *Practitioner.* London, 1888, pp. 321–331.

*Koetsnitz, A.*—"Ein Fall von Actinomykose." *Allg. Med. Centr. Ztg.* Berlin, 1888, pp. 725–728.

*Partsch, E.*—"Die Actinomykose des Menchen vom kleinischen Standpunkt besprochen." *Klin. Vortage.* Leipz., 1888, pp. 306–07.

*Schuchardt, B.*—"Ueber das Vorkommun von Actinomykose beim Elephanten in Indien und uber die Identitat des Madura Fusses mit Actinomykose." *Cor. Bl. der Allg. Arztl. Ver. von Thieringen.* Weimar, 1888, p. 376.

## Figures and Tables

**Figure f1:**
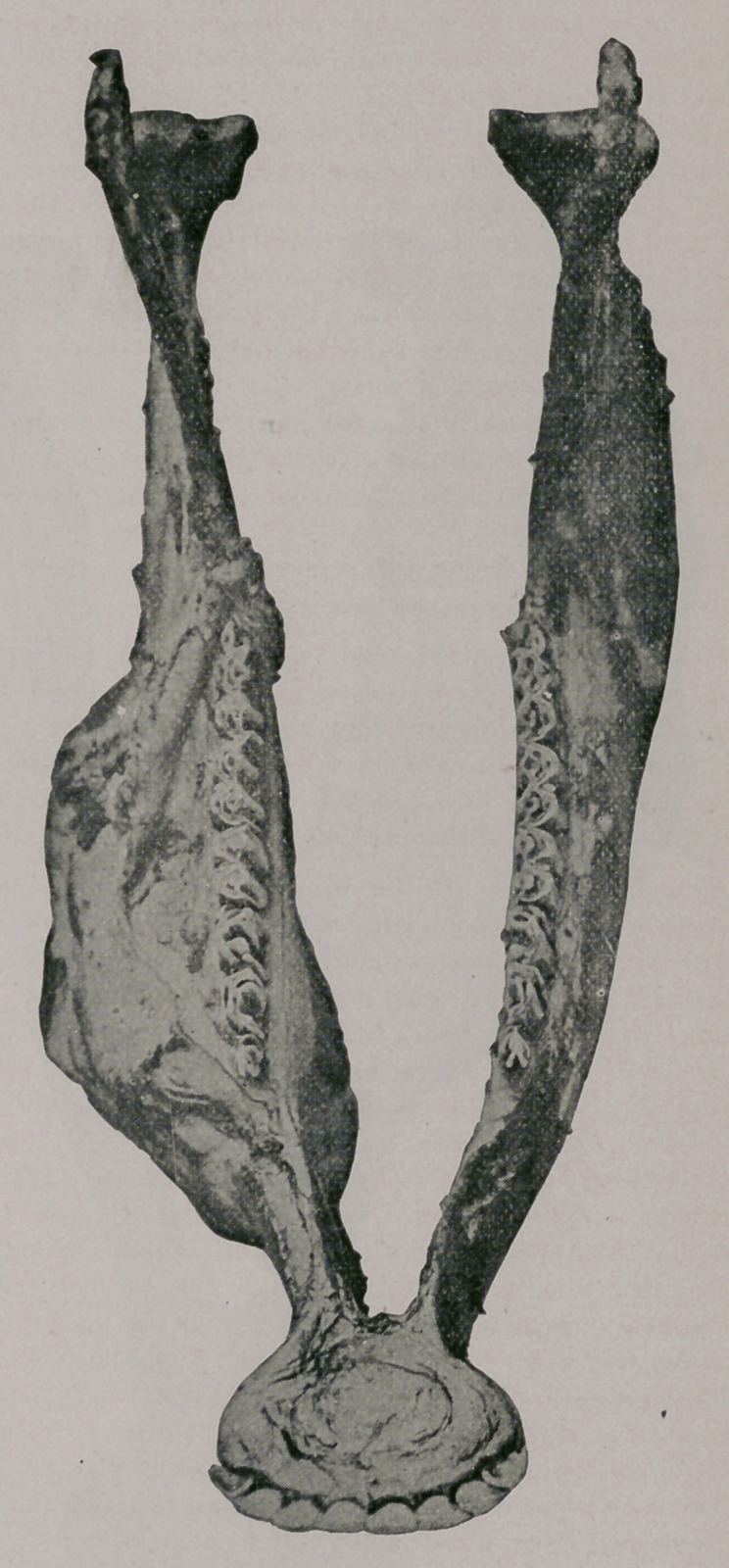


**Figure f2:**
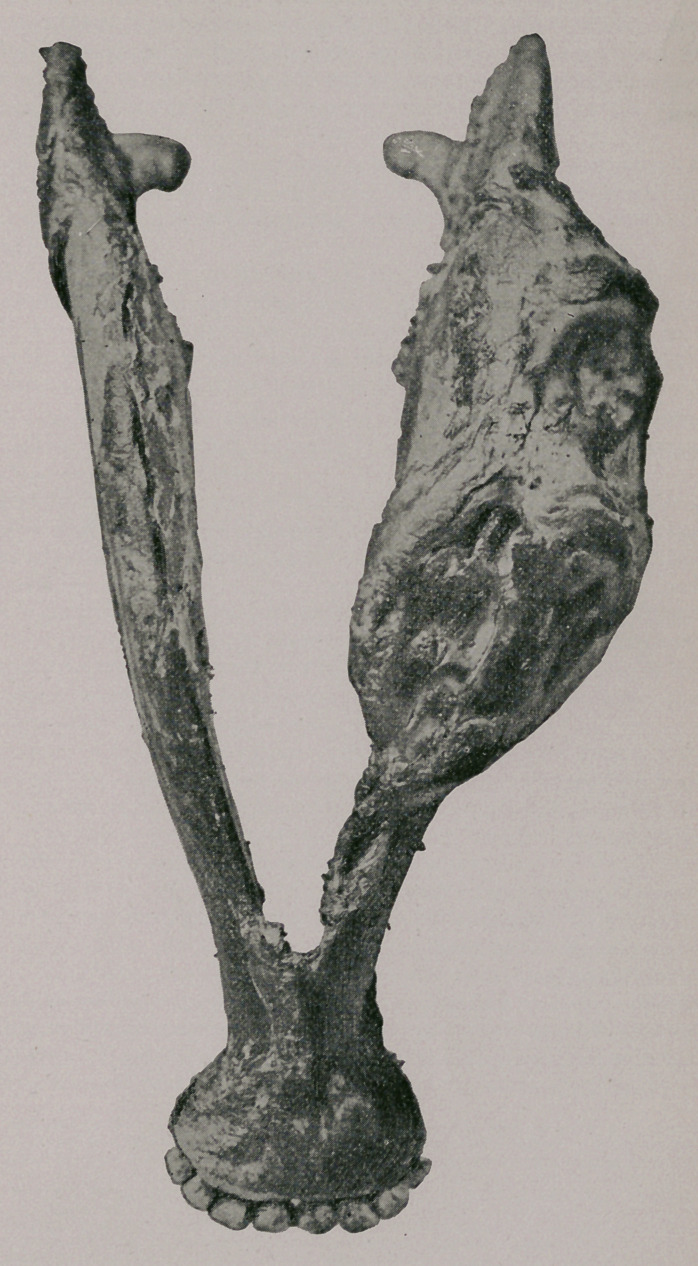


**Figure f3:**